# MR‐based treatment planning in radiation therapy using a deep learning approach

**DOI:** 10.1002/acm2.12554

**Published:** 2019-03-12

**Authors:** Fang Liu, Poonam Yadav, Andrew M. Baschnagel, Alan B. McMillan

**Affiliations:** ^1^ Department of Radiology School of Medicine and Public Health University of Wisconsin Madison WI USA; ^2^ Department of Human Oncology School of Medicine and Public Health University of Wisconsin Madison WI USA

**Keywords:** brain tumor, convolutional neural network, deep learning, MRI, MR‐only treatment planning, radiotherapy

## Abstract

**Purpose:**

To develop and evaluate the feasibility of deep learning approaches for MR‐based treatment planning (deepMTP) in brain tumor radiation therapy.

**Methods and materials:**

A treatment planning pipeline was constructed using a deep learning approach to generate continuously valued pseudo CT images from MR images. A deep convolutional neural network was designed to identify tissue features in volumetric head MR images training with co‐registered kVCT images. A set of 40 retrospective 3D T1‐weighted head images was utilized to train the model, and evaluated in 10 clinical cases with brain metastases by comparing treatment plans using deep learning generated pseudo CT and using an acquired planning kVCT. Paired‐sample Wilcoxon signed rank sum tests were used for statistical analysis to compare dosimetric parameters of plans made with pseudo CT images generated from deepMTP to those made with kVCT‐based clinical treatment plan (CTTP).

**Results:**

deepMTP provides an accurate pseudo CT with Dice coefficients for air: 0.95 ± 0.01, soft tissue: 0.94 ± 0.02, and bone: 0.85 ± 0.02 and a mean absolute error of 75 ± 23 HU compared with acquired kVCTs. The absolute percentage differences of dosimetric parameters between deepMTP and CTTP was 0.24% ± 0.46% for planning target volume (PTV) volume, 1.39% ± 1.31% for maximum dose and 0.27% ± 0.79% for the PTV receiving 95% of the prescribed dose (V95). Furthermore, no significant difference was found for PTV volume (*P *= 0.50), the maximum dose (*P *= 0.83) and V95 (*P *= 0.19) between deepMTP and CTTP.

**Conclusions:**

We have developed an automated approach (deepMTP) that allows generation of a continuously valued pseudo CT from a single high‐resolution 3D MR image and evaluated it in partial brain tumor treatment planning. The deepMTP provided dose distribution with no significant difference relative to a kVCT‐based standard volumetric modulated arc therapy plans.

## 1 INTRODUCTION

In recent years there have been many efforts to develop Magnetic Resonance Imaging (MRI)‐based treatment planning methods that avoid auxiliary computed tomography (CT) for radiation therapy treatment planning.^1^ MRI provides superior soft tissue contrast compared to CT which makes it an excellent image modality to delineate accurate boundaries for targeted treatment regions to deliver the most desirable dose distribution.^2^, ^3^ In addition, image techniques that do not administer ionizing radiation, such as MRI, are desirable for pursuing reduced treatment dose to patients.

A key challenge for MRI‐based treatment planning is the lack of a direct approach to obtain electron density for dose calculation. Unlike conventional CT‐based treatment planning where additional acquired CT images can be scaled to a photon attenuation map (μ‐map),^4^, ^5^ MRI does not provide linear image contrast and is limited in achieving positive contrast in bone (the highest attenuating tissue). Therefore, no straightforward conversion from MR images to a μ‐map is available for compensating dose calculation. Additionally, other challenges include the presence of MR image artifacts as a result of a relatively complex image formulation and potentially long scan time in contrast to CT scans.

Given the importance of an accurate μ‐map to enable accurate dose calculation in treatment planning, the development of novel approaches to generate pseudo CTs or synthetic CTs from MRI is an actively studied topic.^6^, ^7^, ^8^, ^9^ State‐of‐the‐art approaches can be roughly classified into two main categories: image intensity‐based and atlas‐based.^1^ The typical intensity‐based approach is to utilize individual or combined T1‐weighted, T2‐weighted, and water/fat separated MR sequences that estimate tissue compartments with a single or multiple acquisition.^1^ These images are then further processed to directly assign Hounsfield Unit (HU) values to air, fat, lung, and water compartments^10^ or to estimate the continuously valued HU to various tissues by MR signal conversion model.^11^ However, because bone cannot be visualized with positive contrast on conventional MR imaging approaches, bone is typically challenging to estimate in these approaches. Specialized MRI acquisitions using an ultrashort echo time (UTE) or zero echo time can be implemented to allow the measurement of the rapidly decaying MR signal in bone tissue to estimate bone. Unfortunately, most UTE acquisitions are challenging to integrate into clinical workflows as a result of technical difficulties in implementation, require a relatively long scan time, and have limited availability across different vendor platforms. Additionally, even with advanced UTE acquisitions, bony structure and air often remain difficult to distinguish and pose errors in consecutive attenuation calculation. In addition, partial volume effects and signal inhomogeneity due to RF pulse and receive coil arrays are additional confounding factors that influence the accuracy and precision of segmentation‐based approaches in MR.

Atlas‐based approaches for treatment planning utilize registration and spatial normalization of a population‐based CT image template to acquired MR images to estimate the location and geometry of tissue types.^12^, ^13^ A particular advantage of these techniques is that an existing, clinically useful MRI series can be used as the input dataset, eliminating the need for an extra MR acquisition particular for treatment planning. However, this process is highly dependent upon the accuracy of image registration where the patient anatomy must be appropriate for the atlas used. Therefore, atlas‐based approaches may suffer when utilized in subjects with abnormal anatomy such as missing tissues or the presence of surgical implants.

Deep learning utilizing convolutional neural networks (CNN) have recently been applied to medical imaging with successful implementations for a wide range of applications.^14^ A study in Ref^15^ demonstrated the feasibility of pseudo CTs generation using MR images with a deep CNN model. The deep learning method compared favorably with an atlas‐based method for generating pseudo CTs with high accuracy and efficient computation speed at test time. In PET/MR, one pilot study^16^ utilized deep learning to enable accurate generation of pseudo CTs from a single acquisition of T1‐weighted MRI image acquired in standard clinical brain protocol. In this study, a deep CNN model was designed to classify tissues on the MRI images after training with registered kilovoltage CT (kVCT) images. As a result, three‐discrete labels were assigned to soft tissue, air and bone in the generated pseudo CTs that delivered accurate PET/MR attenuation correction with significantly reduced error in reconstructed PET images compared with existing segmentation‐based and atlas‐based methods.^16^ In one recent study from the same group, the deep learning approach was applied in combination with an advanced UTE sequence, which achieved reliable and accurate tissue identification for bone in PET/MR attenuation correction in brain imaging.^17^ Another recent study demonstrated excellent performance utilizing deep learning generated pseudo CTs for PET/MR attenuation correction in pelvis.^18^

The purpose of this study was to further evaluate the efficacy and efficiency of deep learning generated pseudo CTs in the application of treatment planning in radiation therapy on brain tumor patients. Specifically, we evaluated an MRI‐based treatment planning approach, deepMTP, which allows generation of a continuously valued pseudo CT from a single MRI acquisition from clinical protocol using deep CNN model. To the best of our knowledge, this study is one of the pilot studies to implement deep learning generated pseudo CTs into the workflow of treatment planning and to evaluate the accuracy and robustness of such approach for dose calculation accuracy in clinical cases of radiotherapy for brain metastases. While other studies have demonstrated techniques for the generation of pseudo CTs with hypothesized applications for radiotherapy treatment planning, none have evaluated their efficacy in evaluating clinical radiotherapy treatment plans using the generated pseudo CT images.

## 2 MATERIALS AND METHODS

### 2.A Convolutional neural network architecture

**Figure 1:**
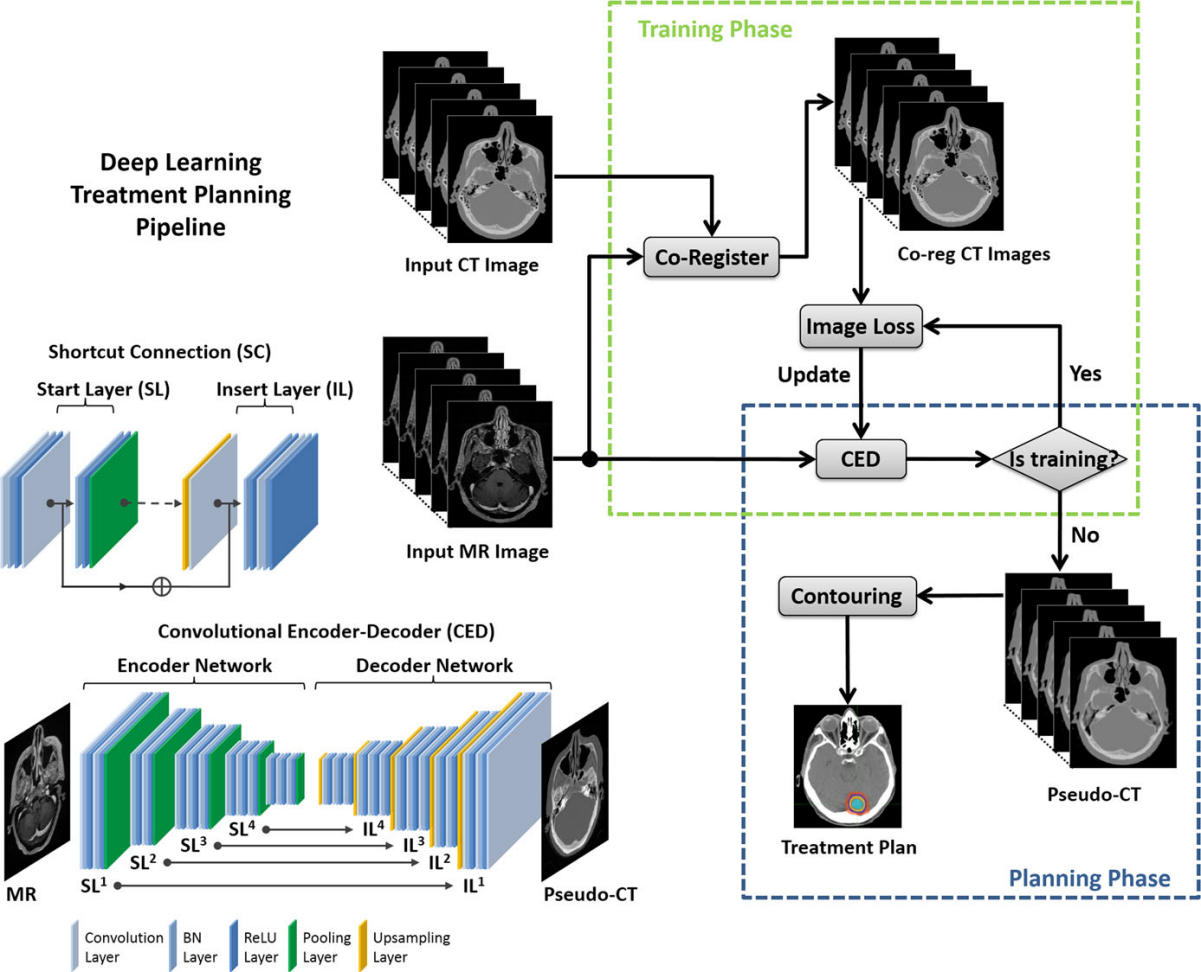
Schematic illustration of deepMTP pipeline. The convolutional encoder‐decoder (CED) network is used to convert MR images into pseudo CT images. This network consists of a combined encoder network (VGG16) and decoder network (reversed VGG16) with multiple symmetrical shortcut connection (SC) between layers. The insertion of SC follows the strategy of full preactivation deep residual network. The deepMTP process consists of a training phase to optimize the CED network and a planning phase to generate pseudo CTs for new MR data using trained and fixed CED network. This figure is adapted from the Figure 1 of the Ref. 16 with permission to be used in this paper.

Inspired by the network design in Ref., 16, 17 we utilized the deep convolutional encoder‐decoder network structure shown in Fig. 1, which is capable of mapping pixel‐wise image intensity from MRI to CT in multiple image scales. The basic framework of this type of network was built based on structures that perform well in natural image object recognition^19^ and MRI segmentation for various tissues.^20^, ^21^, ^22^, ^23^, ^24^ The network consisted an encoder network directly followed by a decoder network, where the encoder network uses a set of combined 2D convolution filtering, batch normalization,^25^ ReLU activation,^26^ and max‐pooling to achieve image feature extraction for unique spatial invariant input image features. The decoder network takes the output of the encoder network and combines extracted image features in multiple resolution scale to generate targeted high‐resolution image output through an image upsampling process. The encoder uses the same 13 convolutional layers from the VGG16 network^27^ originally designed for image recognition and later tested in multiple image segmentation tasks.^16^, ^20^ The decoder is applied directly after the encoder network and features a reversed VGG16 network structure with the max‐pooling layers replaced by corresponding upsampling layers. In Ref., 16 the pseudo CT generation was treated as a semantic segmentation problem for multiple tissue classes in MR images. More specifically, a multiclass softmax layer was inserted into the final layer of decoder network and the model was optimized with multiclass cross‐entropy image loss which yields a pixel‐wise discrete label output matching the input MR image resolution. The different fixed HU values were assigned to different tissue compartments based on the discrete label to create discrete pseudo CT model. In this study, we substituted the softmax layer with a 2D convolutional layer and optimized image loss using a mean square error cost function. The pseudo CT generation was treated as a signal regression problem for converting MR contrast to CT contrast. More specifically, instead of outputting tissue classes as in Ref., 16 the CNN in this study directly outputted pseudo CTs and the actual pixel‐wise HU values of pseudo CTs were optimized against the real CT values to minimize the contrast difference. As a result, an output of pseudo CTs with continuously valued HU number is enabled in contrast to the output of discrete tissue labels in Ref. 16.

Additionally, like the popular U‐Net,^28^ shortcut connections (SC) were added between the encoder and decoder network to enhance the mapping performance of the encoder‐decoder structure. The added SC are advantageous in preventing excess image detail loss during the max‐pooling process of the encoder in deep CNN networks.^29^, ^30^ A total of four SC were created between the network layers by following the full preactivation method described in the deep residual network configuration^30^ and one additional shortcut connection was also generated from the input image directly to the output image. The detailed structure of the proposed networks is schematically illustrated in Fig. 1.

### 2.B deepMTP procedure

The proposed deepMTP procedure consisted of two independent phases for training retrospective MRI and coregistered CT data and for generating pseudo CTs using a fixed network in the treatment planning phase. As also shown in Fig. 1, in the training phase, 3D CT images were first coregistered to MR images using a combined rigid Euler transformation and nonrigid B‐spline transformation using the Elastix image registration tool^31^ following the method described in Ref. 32. Specifically, a 4‐level multiple resolution strategy, 32 histogram bin similarity measurement and a total of 1500 iteration were performed. An optimization metric combining localized mutual information with the bending energy penalty term was used for nonrigid registration. For each training dataset, MR and coregistered CT images were first offset to positive values and then scaled by pixel intensity of 5000 and 2000 (HU), respectively, to ensure the similar dynamic range. Then the 3D MR and CT volume data were input into the encoder network as a stack of 2D axial images. The network weights were initialized using the scheme described in Ref. 33 and updated using the ADAM algorithm^34^ with a fixed learning rate of 0.001. The network was trained in a mini‐batch manner with 16 images in each batch and with total iteration steps corresponding to 50 epochs for the training data. The changes in training loss were observed to be less than 0.3% within the last 10 epochs, indicating a training convergence. Once training was finished, the network weights were fixed to the values at the epoch with the lowest image loss during all training procedure. The fixed network was then used to generate continuously valued pseudo CT images from newly acquired MR images and proceeded to simulated treatment planning.

All training and evaluation were performed on a 64‐bit Linux workstation. Computing hardware included an Intel Xeon W3520 quad‐core CPU, 32 GB RAM, and two Nvidia GeForce GTX 1080 Ti graphic cards with a total 7168 cores and 22GB GPU RAM. The deepMTP framework was implemented in a hybrid computing environment involving Python (version 2.7, Python Software Foundation, Wilmington, DE, USA) and MATLAB (version 2013a, MathWorks, Natick, MA, USA). The image pre and postprocessing was implemented using MATLAB program and the deep learning network was configured and coded using the Keras package with Tensorflow as the computing backend.^35^

### 2.C Image datasets for deepMTP

Analysis was performed in compliance with Health Insurance Portability and Accountability Act regulations and with approval from our Institutional Review Board (IRB). All subject data were obtained with a waiver of consent under IRB approval. Training of the proposed deepMTP network was performed on retrospective head images from 40 subjects who underwent both a high‐resolution T1‐weighted postcontrast 3D MR scan and a noncontrast CT scan on the same day for evaluation of acute stroke. Subjects had a median age of 61 (range: 21–91) with 22 males and 18 females. Gadobenate dimeglumine (MultiHance; Bracco Diagnostics, Princeton, NJ, USA) contrast was administered at 0.1 mmol/kg. The MR images were acquired on two 1.5 T scanners (Signa HDxt, MR450w; GE Healthcare, Waukesha, WI, USA) with the following parameters: T1 BRAVO pulse sequence, 0.46–0.52 mm transaxial voxel dimensions, 1.2 mm slice thickness, 450 ms inversion time, 8.9–10.4 ms repetition time, 3.5–3.8 ms echo time, 13° flip angle, with an 8‐channel receive‐only head coil. There is a total number of 5840 MR image slices for the 40 subjects. Likewise, training CT images in these same subjects were acquired on three scanners (Optima CT 660, Discovery CT750 HD, Revolution GSI; GE Healthcare, Waukesha, WI, USA) with the following acquisition/reconstruction settings: 0.43–0.46 mm transaxial voxel dimensions, 1.25–2.5 mm slice thicknesses, 120 kVp, automatic exposure control with GE noise index of 2.8–12.4, and 0.53 helical pitch.

Evaluation of the trained model was performed on 10 randomly selected patients with brain tumor, treated with Fractionated Stereotactic Radiotherapy. Selected cases had a median age of 62 (range: 47–74) which included three males and seven females. The lesion location for these patients varied from cerebellar, parietal, and frontal regions of the brain. A high‐resolution T1‐weighted postcontrast 3D MR scan was performed for diagnostic with the above‐mentioned protocol on two 1.5 T scanners (Signa HDxt and MR450w; GE Healthcare, Waukesha, WI, USA). There is a total number of 2720 MR image slices for the 10 patients. The kVCT images for the clinical treatment plan were acquired on Siemens CT scanner (Somatom Definition Edge; Siemens Healthcare, Erlangen, Germany). To minimize motion and reproduce the setup for daily treatments patients were simulated wearing a head mask, holding a o‐ring in their hand resting on chest and legs resting on cushion. Acquisition/reconstruction settings: 0.98 mm transaxial voxel dimensions, 2.5 mm slice thicknesses, 120 kVp, effective mAs 425, and 1 helical pitch. All scans were exported to MIM (version 6.7, MIM Software Inc., Cleveland, OH, USA) for target and organ at risk (OAR) segmentation by radiation oncologist. Finally, CT scans and segmented target and OAR were exported to Pinnacle (Philips, Fitchburg, WI, USA) for planning.

### 2.D Evaluation of Pseudo CTs

Evaluation of pseudo CT accuracy was performed on above mentioned 10 subjects who were not involved in training phase. We used the Dice coefficient, a similarity measure ranging from 0 to 1 that describes the overlap between two labels, to calculate the classification accuracy for soft tissue, bone, and air, where pseudo CT generated from deepMTP and the ground truth (kVCT image) were compared. For calculation of Dice coefficients, the continuously valued pseudo CT and planning kVCT images were discretized by thresholding as following: bone if HU >300, air if HU <−400, otherwise soft tissue. Additionally, the mean absolute error (MAE) within the head region was also evaluated between pseudo CT and kVCT for each subject to elucidate the overall pixel‐wise image error.

### 2.E Treatment planning

Evaluation of the feasibility of treatment planning using pseudo CT was performed on 10 subjects. The T1‐wegithed postcontrast 3D MR images were exported to MIM along with kVCT. The CT images were registered to the MR image (and pseudo CT) in MIM for tumor delineation. Apart from planning target volume (PTV), brainstem, chiasm, lens, optical nerves, cochleas, and spinal cord were also contoured for the plan optimization and evaluation. Prescribed dose varied from 24–32 Gy in 3–8 fractions. A volumetric modulated arc therapy plans was generated with a 6 MV photon beam using 2–5 arcs with pseudo CT and kVCT (referred to as CTTP thereafter) respectively. All plans were generated on Pinnacle (Philips, Fitchburg, WI, USA), keeping the same optimization parameters. The dose was calculated with an adaptive convolution superposition algorithm with a dose matrix resolution of 0.2 × 0.2 × 0.2 cm^3^ or 0.25 × 0.25 × 0.25 cm^3^. All clinical treatment plans were checked for the delivery quality assurance (DQA) prior to treatment. Thus, DQA plans were generated on the treatment planning station for all study cases on ScandiDos Delta4. It was expected that at least 95% of all measured points pass the defined gamma criteria (TG 119).

### 2.F Evaluation of dose distribution

The calculated dose was exported to MIM for plan comparison using dose volume histogram (DVH) and isodose distribution. For PTV, the maximum dose and the PTV receiving 95% of the prescribed dose (V95) were recorded for dosimetric comparison. Absolute percentage differences between deepMTP and CTTP in PTV for maximum dose, and V95 were calculated after normalizing to CTTP parameter values. Plan quality for the clinical use was evaluated by checking if 95% of the PTV receives 100% of the prescribed dose. Additionally, nonparametric paired‐sample Wilcoxon signed rank sum tests were used to perform pairwise comparison for dosimetric parameters between deepMTP and CTTP. Statistical analysis was performed using MATLAB (version 2013a, MathWorks, Natick, USA) with statistical significance defined as a *P *< 0.05.

## 3 RESULTS

For the deepMTP procedure, the training phase required approximately 2.5 hrs of computational time in our dataset, whereas generating a single pseudo CT image using the trained model and input MR images required roughly 1 minute.

**Figure 2:**
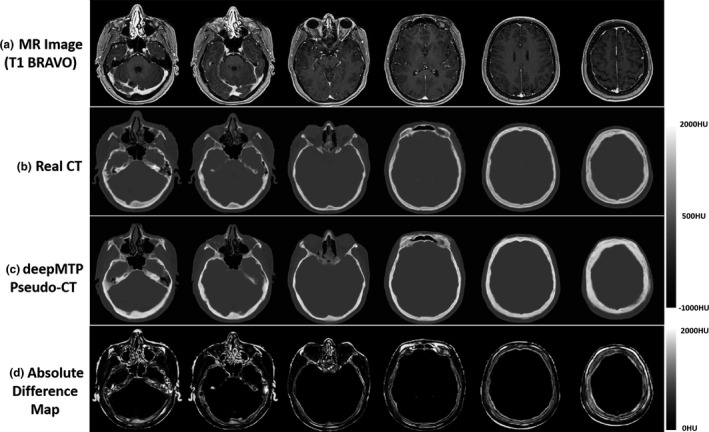
Example pseudo CT images from deepMTP from a 47‐year‐old female patient with right cerebellar tumor. Multiple slices from (a) the input 1.5 T T1 BRAVO MR image, (b) the acquired CT, (c) the pseudo CT generated using deepMTP, and (d) the absolute difference map.

An example of an acquired 1.5 T MRI, actual CT, and pseudo CT for a 47‐year‐old female patient with right cerebellar metastasis is demonstrated in Fig. 2. As shown, deepMTP was able to accurately transfer MR image contrast into CT images with clearly identified air, brain soft tissue, and bone highly similar to that of kVCT images. Evaluation of the Dice coefficient in 10 brain metastases cases comparing the output tissue mask from the pseudo CT to the kVCT mask was high for air: 0.95 ± 0.01, soft tissue: 0.94 ± 0.02, and bone: 0.85 ± 0.02. The MAE between pseudo CT and kVCT was 75 ± 23 HU for all 10 subjects.

**Figure 3:**
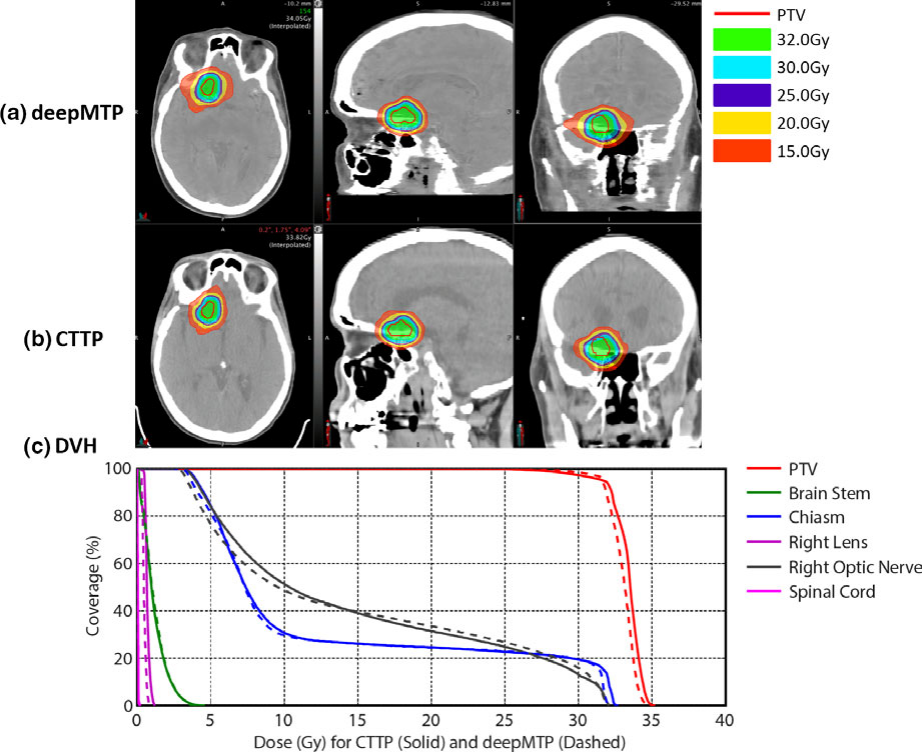
An example of a 54‐year‐old female patient with right frontal brain tumor adjacent to chiasm and right optic nerves shows similar isodose lines (a) and (b) and DVH (c) between deepMTP and CTTP.

**Figure 4:**
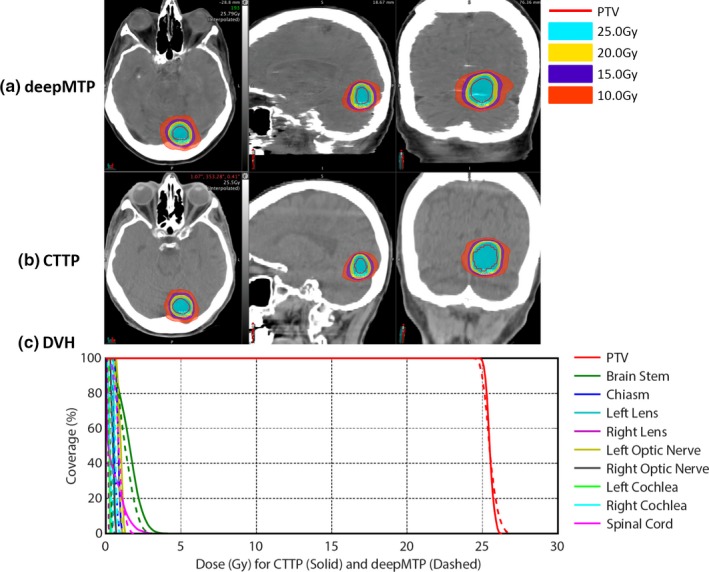
An example of a 74‐year‐old male patient with inferior brain tumor near occipital bone shows similar isodose lines (a) and (b) and DVH (c) between deepMTP and CTTP.

**Figure 5:**
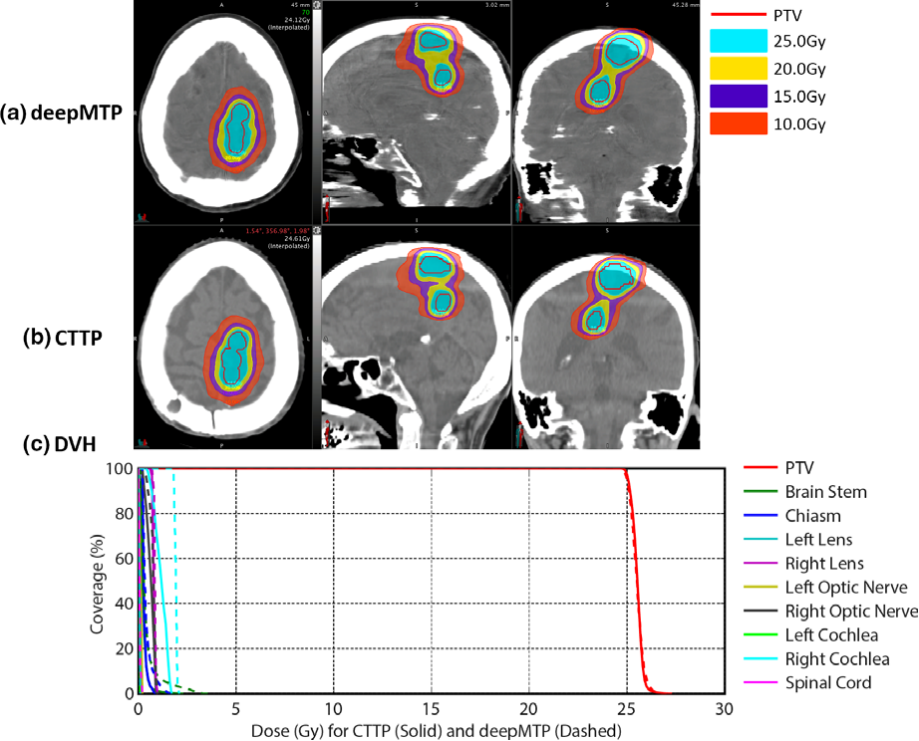
An example of a 74‐year‐old male patient with a large superior brain tumor shows similar isodose lines (a) and (b) and almost identical PTV dose curves in DVH (c) between deepMTP and CTTP.

The absolute percentage differences for deepMTP in comparison to CTTP was 0.24% ± 0.46% for PTV, 1.39% ± 1.31% for maximum dose, and 0.27% ± 0.79% for V95. There was no significant difference between CTTP and deepMTP for PTV (*P *= 0.50), maximum dose (*P *= 0.83) as well as V95 (*P *= 0.19) in the 10 tested subjects (Table 1). The dose distribution and DVH for three clinical cases were illustrated in Figs. 3, 4, 5 representing small and large lesions at different location of the brain including soft tissue and bony area.

**Table 1:** Mean and standard deviation of dosimetric parameters and their absolute percentage differences of 10 patients using deepMTP and CTTP, respectively, and *P*‐values from nonparametric paired‐sample Wilcoxon signed rank sum test comparing deepMTP and CTTP

Dosimetric parameters	deepMTP	CTTP	Difference (%)	Wilcoxon *P*‐value
PTV (cc)	20.76 ± 31.82	20.77 ± 31.86	0.24 ± 0.46	0.50
Maximum dose (Gy)	30.79 ± 4.29	30.76 ± 4.51	1.39 ± 1.31	0.83
V95 (%)	99.43 ± 1.16	99.65 ± 0.96	0.27 ± 0.79	0.19

An example of a 54‐year‐old female patient with right frontal brain tumor adjacent to chiasm and right optic nerves is shown in Fig. 3. The MAE between pseudo CT and kVCT was 72 HU for this subject. deepMTP provided a treatment plan with similar PTV and isodose lines around the tumor region compared with CTTP in the fused MR and CT images [Fig. 3(a) and 3(b)]. The plan was designed to avoid adjacent chiasm and optic nerves. The DVH [Fig. 3(c)] showed highly similar dose curves for the PTV, chiasm and right optic nerve between CTTP (solid line) and deepMTP (dashed line). The other OARs received a clinical relevant low dose as desired for the clinical treatment plan.

An example of a 74‐year‐old male patient with an inferior brain tumor near occipital bone is demonstrated in Fig. 4. The MAE between pseudo CT and kVCT was 95 HU for this subject. Both the PTV and isodose lines showed a high similarity between CTTP and deepMTP [Fig. 4(a) and 4(b)] in the tumor region. There was negligible difference for PTV in DVH dose curve between CTTP and deepMTP [Fig. 4(c)] and a low dose was received by other OARs as planned.

Another example of a 74‐year‐old male patient with a large superior brain tumor is demonstrated in Fig. 5. The MAE between pseudo CT and kVCT was 69 HU for this subject. The tumor represented an elongated oval shape for planning. Both CTTP and deepMTP delivered highly similar PTV and isodose lines covering the tumor region [Fig. 5(a) and 5(b)]. The PTV dose curves in DVH of this subject showed almost identical results between CTTP and deepMTP [Fig. 5(c)]. The other OARs received considerable low dose as planned.

All test cases passed the clinical planning dosimetry constraints, indicating acceptable treatment planning performance for deepMTP relative to conventional CTTP. Individual set of dosimetric data for all OARs was reviewed and no reportable dose difference was found for test cases. The subject shown in Fig. 3 had the worst dosimetric difference between CTTP and deepMTP, whereas the subjects shown in Figs. 4 and 5 had typical dosimetric comparison results between CTTP and deepMTP.

The absolute measurements were compared to the planned using global 3%/3 mm gamma analysis. Average global 3%/3 mm Gamma analysis pass rates of 99.2% were recorded when typical clinical dose calculation parameters were used for all test cases.

## 4 DISCUSSION

For the 10 subjects evaluated using deepMTP, there was no significant dosimetric difference in dose to OAR and maximum dose, and V95 for PTV suggesting a high level of performance. All pseudo CT plans meet clinical planning dose constraints to OAR. In comparison to other approaches for pseudo CT generation in the brain and applied to RT planning, the performance of the proposed deepMTP compares favorably. The measured MAE of pseudo CT generation of 75 ± 23 HU and bone Dice coefficient of 0.85, is similar or better than previously reported techniques^36^, ^37^, ^38^, ^39^, ^40^ (with MAE ranging from 85 to 150 HU and bone Dice coefficient ranging from 0.74 to 0.85). Dosimetrically, results are generally similar and appear to be appropriate for clinical use; however, different metrics have been used to assess dose accuracy across different approaches.

One of the primary advantages of the deepMTP approach is the use of a clinically relevant MRI acquisition. The postcontrast T1‐weighted image utilized herein is a standard sequence that is expected to be utilized clinically. Other proposed approaches utilize specialized techniques such as ultrashort time echo (UTE) which provide positive MRI signal contrast in bone. UTE sequences often provide limited additional clinical value over conventional acquisitions, and require non‐Cartesian reconstruction algorithms and specialized system calibration for optimal performance.

An additional advantage of the deep learning approach is the simplification provided to the RT planning workflow. Specifically, after training the deep learning network, the acquired MR input images require minimal preprocessing, and pseudo CT images can be generated in less than a minute which is highly compatible with clinical workflows. Compared to conventional approaches where the soft tissue target (provided by MRI) is spatially coregistered to a planning CT using software tools,^41^, ^42^, ^43^, ^44^, ^45^, ^46^ the utilization of deepMTP would enable planning to occur in the pixel space of the MR imaging data without the need for image coregistration. Note that geometric accuracy for the utilized MR scanners must be properly calibrated to ensure minimal distortion in the acquired MR images.^47^, ^48^, ^49^, ^50^ In comparison to MR‐only approaches that utilize atlas‐based approaches, deep learning‐based approaches can be computed much more rapidly and are expected to be capable of including individual subject variations in properly trained models.

Future study is necessary to determine limitation of deepMTP approach based on anatomical abnormalities compared to purely template‐driven MR‐only approaches. However, this remains to be determined on large cohorts of clinical patients. While deepMTP provided a high Dice coefficient for classifying air, soft tissue, and bone in the brain dataset, small amounts of tissue misclassification was observed in pseudo CTs near complex structures such as the interface of air and bone in sinus. However, this small misclassification seems to only have negligible influence on treatment planning accuracy for the brain tumors. However, misclassification might have greater dosimetric impact on other sites such as head and neck, lung and prostate as indicated in previous studies.^39^, ^51^, ^52^

Note that deep learning methods likely require anatomy and/or region‐specific training to maximize performance. In our experience, the use of a deep learning model trained in the head will not be applicable to other regions of the body. This should be expected as different MR imaging sequences are used for diagnostic imaging in different regions the body (e.g., the T1 BRAVO used for high‐resolution brain imaging is not acquired in other body regions). Evaluation elsewhere in the body will require retraining of the model with high‐quality (i.e., high‐resolution and artifact‐free) images from each respective region. However, while new input data would be required to train a model, it is expected that the architecture used herein for deepMTP would be directly applicable to other regions. This supports the use of transfer learning,^53^, ^54^ where a trained model in the head could be used to reduce the amount of training data in another body region if similar image contrast (e.g., T1‐weighted) is obtained.

MRI has been increasingly incorporated into the planning and delivery of radiation treatment.^1^, ^55^, ^56^, ^57^ Given that nearly half of all cancer patients receive radiation during their treatment, there are a substantial number of patients likely to benefit from improved approaches of integrating MRI into RT planning. The further development of MR‐only approaches will help to reduce radiation dose from kVCT. This is advantageous in particular for treatment of pediatric and pregnant cases where radiation dose reduction is the primary goal. Future applications of MR‐only treatment planning will be improved if the MR scan can be performed in the radiation treatment position, which will require development of more MR compatible setup equipment and improved capabilities for MR imaging in a large field of view. Furthermore, recent advances in technology have resulted in therapy devices that combine MR scanners with RT devices (e.g., ^60^Co IGRT^58^, ^59^, ^60^, ^61^ and linear accelerators^62^, ^63^, ^64^) which further support the use of MRRT and other advanced methods to improve and augment therapy delivery, such as interfraction assessment of therapy response and inter‐ and intrafraction adaptation of therapy plans.^65^, ^66^ These systems also allow real‐time imaging during treatment, which may prevent geometric tumor miss and allow for a smaller PTV to be used. This capability is particularly advantageous in the lung and upper abdominal cancers were respiratory tumor motion must be taken into account. Therefore, future development of approaches to extend deepMTP to other regions of the body will have additional impact.

## 5 CONCLUSION

We have shown that deep learning approaches applied to MR‐based treatment planning in radiation therapy can produce comparable plans relative to CT‐based methods. The further development and clinical evaluation of such approaches for MR‐based treatment planning has potential value for providing accurate dose coverage and reducing treatment unrelated dose in radiation therapy, improving workflow for MR‐only treatment planning, combined with the improved soft tissue contrast and resolution of MR. Our study demonstrates that deep learning approaches such as deepMTP, as described herein, will have a substantial impact on future work in treatment planning in the brain and elsewhere the body.

## CONFLICT OF INTEREST

All authors have no conflict of interest to disclose.
